# A Review of the Advancements in Targeted Therapies for Breast Cancer

**DOI:** 10.7759/cureus.47847

**Published:** 2023-10-28

**Authors:** John Kessellie Jallah, Tuward J Dweh, Ashish Anjankar, Ogiza Palma

**Affiliations:** 1 Department of Biochemistry, Datta Meghe Institute of Higher Education and Research, Wardha, IND; 2 Department of Biotechnology, C.V. Raman Global University, Bhubaneswar, IND; 3 Department of Biochemistry, Jawaharlal Nehru Medical College, Datta Meghe Institute of Higher Education and Research, Wardha, IND

**Keywords:** breast carcinoma, targeted cancer therapy, hormone receptor, triple-negative breast, her2+ breast cancer

## Abstract

Breast cancer, the second-most common and lethal disease in women, poses a severe danger to global health. Breast cancer rates continue to climb despite advances in medical technology. Predictions indicate that by 2040, there will be more than three million new cases yearly. Targeted medicines have experienced a profound transformation in treating breast cancer, allowing for individualized strategies that lessen side effects and improve patient outcomes. This thorough analysis gives a rigorous investigation of current developments in breast cancer-targeted treatments. It carefully examines several subtypes, including hormone receptor-positive (HR+), HER2-positive (HER2+), and triple-negative breast cancer (TNBC), recognizing the illness' fundamental variety. It offers specialized treatment plans catered to each subtype's particular traits. The review also examines how precise genetic abnormalities like BRCA1/2 and PIK3CA mutations and molecular profiling facilitate therapy selection. Monoclonal antibodies and small molecule inhibitors are some of the targeted medicines examined in the study. It explains how each of these treatments works and supports its findings with data from clinical trials. It also considers potential new medications and addresses persistent problems, such as resistance mechanisms, chances for combining therapies, and cutting-edge patient classification techniques. This study seeks to give healthcare professionals, researchers, and patients a thorough overview of the recent advancements in breast cancer-targeted therapy by drawing on the opinions of top authorities in the area. The coordinated effort aims to create customized, efficient therapies, eventually bolstering the battle against this powerful illness.

## Introduction and background

Despite continuous scientific advancements, breast cancer stands as a significant and severe disease among women, ranking as the second most prevalent. There has been a notable rise in the incidence of breast cancer over the past four decades [1]. There were around 685,000 new instances of breast cancer globally in 2020, with noteworthy regional differences across various nations and regions [2]. Significantly, breast cancer mortality rates are elevated in nations with strong economies. Projections indicate that by 2040, over one million individuals will succumb to breast cancer, with more than three million people being diagnosed [3]. Cancer remains the prevalent ailment among women and poses the foremost threat to global health, as it leads to cancer-related fatalities, endangering health worldwide. Its influence on public health remains significant despite early identification and treatment advancements. The breast cancer treatment paradigm has changed in recent years due to improved comprehension of the illness's complex molecular and genetic causes [4]. Targeted medicines have become a potent tool in the battle against breast cancer, promising precision medicine and better patient outcomes. This broad study aims to summarize the current knowledge in this crucial field and provide a comprehensive overview of the most recent advancements in breast cancer targeted treatment. Historically, non-selective cytotoxic chemotherapies, radiation, and surgical procedures were the mainstays of breast cancer treatment. Even though these treatments improved patient survival significantly, they frequently came with significant side effects and unpredictable treatment outcomes. A new era in breast cancer therapy was ushered in by the development of targeted medicines, which emphasized customized care by matching therapeutic approaches to the unique molecular features of each patient's tumor [5]. This paradigm change increases therapeutic effectiveness while minimizing treatment-related adverse effects.

Recognizing the disease's variability is essential to comprehending targeted treatments for breast cancer. Various diverse breast cancer subtypes exist; certain clinical traits and molecular profiles distinguish each. Notably, 75% of cases that occur in breast cancer are categorized as hormone receptor (HR)-positive breast cancer fueled by estrogen and progesterone receptors [6]. Endocrine drugs, such as selective estrogen receptor modulators (SERMs) and aromatase inhibitors (AIs), are the primary therapies in this class [7]. Due to the detection of human epidermal growth factor receptor 2 (HER2) gene amplification or excessive membrane expression in a subgroup of breast tumors, trastuzumab and pertuzumab were created as targeted HER2 therapies [8]. Contrarily, due to its aggressive nature and few therapy choices, triple-negative breast cancer (TNBC), characterized by the absence of HR and HER2 receptors, presents a therapeutic hurdle [9].

Immunotherapeutic methods, most notably immune checkpoint inhibitors like pembrolizumab, have proven successful in a few TNBC cases by harnessing the immune system of the patient to combat the tumor [10]. The discovery of biomarkers and molecular profiling has become crucial for directing breast cancer therapy choices. Genetic abnormalities, including BRCA1/2 and PIK3CA mutations, affect treatment options and forecast how patients respond to particular medications. These molecular markers have allowed for more precise treatment planning, which has improved results and reduced unneeded side effects [11]. Monoclonal antibodies, small molecule inhibitors, antibody-drug conjugates, and immunotherapies are all part of the fast-growing landscape of targeted options for breast cancer care. By explicitly targeting HER2-overexpressing cells, monoclonal antibodies like trastuzumab have altered the management of HER2+ breast cancer and raised survival rates. Molecule inhibitors, like palbociclib and ribociclib, have changed the treatment of HR+ metastatic breast cancer by inhibiting CDK4/6 to interfere with cell cycle control [12,13]. Immunotherapies like programmed cell death protein 1 (PD-1) have offered a novel approach to treating breast cancer by enhancing the body's immune response against tumor cells [14,15]. In the following parts of this study, we shall thoroughly investigate the most critical targeted medicines now used in clinical practice. We will describe their mechanisms of action, review the clinical trial data supporting their efficacy, and discuss how they can be utilized to treat various subtypes of breast cancer. We will also explore upcoming changes in therapies, including some in preclinical and early clinical development. We will also discuss the difficulties in maximizing targeted medicines for breast cancer, including problems with acquired resistance mechanisms, the possibility of combination therapy, and creative patient classification methods. This comprehensive study synthesizes a wide range of material, including research findings, clinical trial outcomes, and case studies. It does so by drawing on the expertise of renowned academics and medical professionals in the field. We aim to provide healthcare professionals, researchers, and patients with the knowledge necessary for well-informed decision-making by thoroughly reviewing the most recent developments in targeted therapy for breast cancer. Our combined effort aims to further the continuous search for more individualized and effective therapies, eventually improving the fight against this powerful illness.

## Review

Search methodology

**Figure 1:**
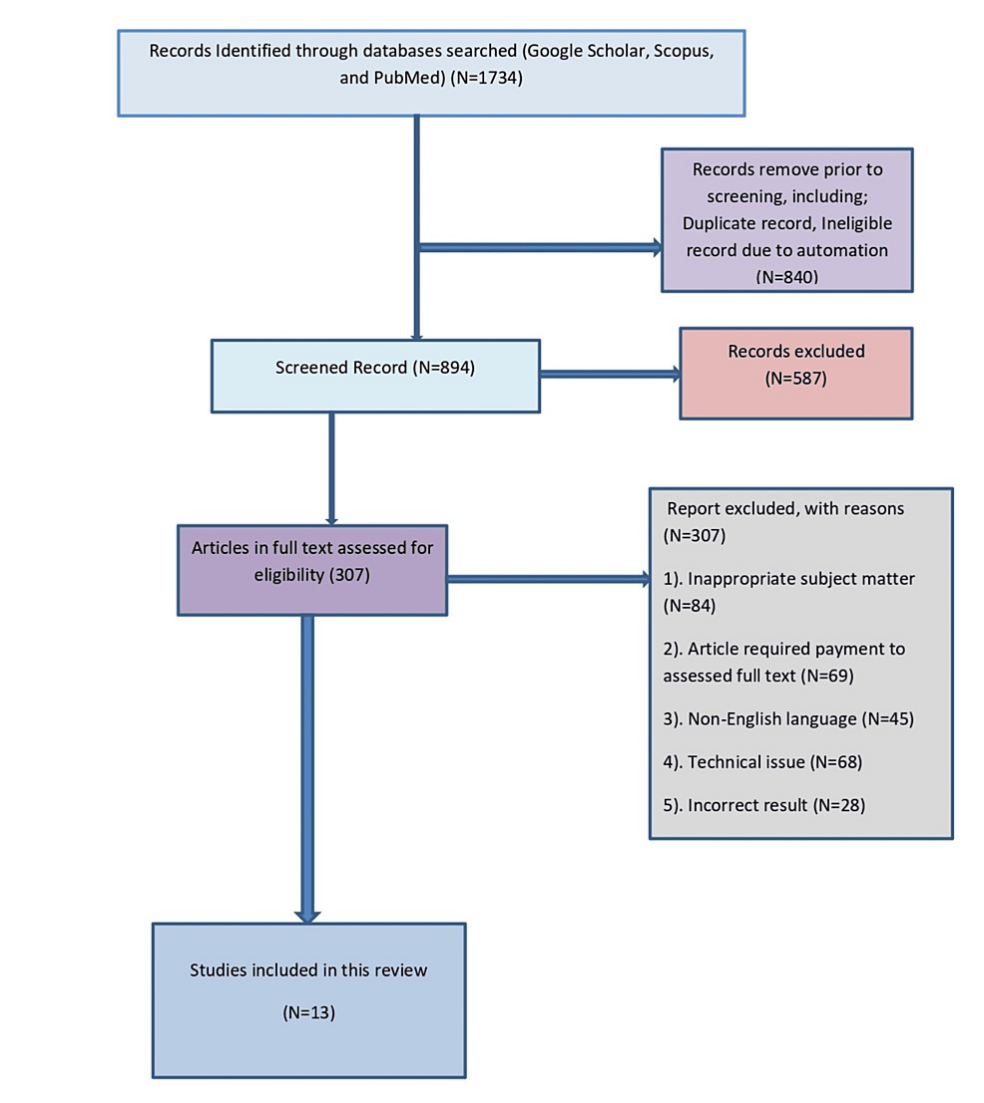
PRISMA flow diagram for inclusion and exclusion criteria PRISMA: Preferred Reporting Items for Systematic Reviews and Meta-Analyses.

For this review article, the researchers conducted an organized investigation and comparative analysis of several breast cancer-related studies. Furthermore, the researchers look at the most recent diagnostic methods, medical discoveries in breast cancer medicines, and improvements in breast carcinoma treatments. Additionally, the researchers started a thorough literature search utilizing keywords like "breast carcinoma," "targeted therapies," "HER2-positive", "hormone receptor," and "triple-negative BC" through various academic journals and search engines like Google Scholar, PubMed, and Scopus. The following are the selection criteria used in this study (Figure 1): (1) targeted therapy; (2) breast cancer; (3) HER2-positive; (4) hormone receptor; (5) metastatic; (6) English language. The following conditions were excluded: (1) inappropriate topic matter; (2) technical issue; (3) article required payment; (4) non-English language. This review also explored advances in Breast cancer therapy procedures, such as surgery, radiation, chemotherapy, and immunotherapy.

Breast cancer: a major global health concern

Breast cancer is challenging for clinicians and researchers since it is categorized based on membrane receptor tumor profile and clinical presentations. Over the years, significant research efforts have been directed at creating customized medicines that can enhance patient outcomes, decrease side effects, and enhance their quality of life. For example, the prognosis of patients with HER2-positive breast cancer has significantly improved as a result of the successful targeting of HER2 overexpression with monoclonal antibodies like trastuzumab (Herceptin) and pertuzumab (Perjeta) [12,16]. These treatments have significantly decreased the chance of recurrence and increased survival. The use of CDK4/6 inhibitors, such as palbociclib, ribociclib, and abemaciclib, to treat HR+ breast cancer is another encouraging advancement in breast cancer targeted therapy [17]. CDK4-6 inhibitors improve median progression-free survival and delay progression of disease, with ribociclib also showing an improvement in overall survival [18,19]. Another significant development is the introduction of immunotherapy as a potential breast cancer treatment strategy. Patients with TNBC may benefit significantly from immunotherapy, chemotherapy, or other targeted therapies. New biomarkers and therapeutic targets are being sought through continuing research in addition to these tailored medicines [14,20]. The identification of genetic abnormalities and changes that promote breast cancer growth has been made possible through genomic profiling. Personalized therapeutics are becoming a reality in breast cancer therapy. An instance includes the utilization of PARP inhibitors in BRCA-mutated breast cancer [21]. Despite the indisputably positive developments in targeted therapy for breast cancer, difficulties still exist. Targeted inherent and acquired therapy resistance remains a significant challenge [22]. Understanding the underlying molecular causes of resistance and creating cutting-edge tactics to combat it are essential. Furthermore, the high price of some targeted medicines creates financial difficulties and concerns regarding equal access to these treatments.

Breast cancer subtypes

Breast cancer mostly affects women and it is a neoplasm that has several different subcategories. Currently, there are three big categories based on tumor profile: (1) hormone receptor-positive (HR+); (2) HER2-positive breast cancer; (3) triple-negative breast cancer [23,24]. Between 70% and 75% of cases may be accurately diagnosed due to the estrogen receptor (ER) [25]. Compared to cases involving ER-negative cases, progesterone receptor (PR) was expressed in more than 50% of those with ER+. Since ER regulates PR activities, having normal PR is essential for the proper functioning of ER. Conversely, the biomarkers of ER and PR are highly expressed in the cancerous cells of the breast and employed in both diagnosis and prognosis [26].

Hormone receptor-positive (HR+)

Progesterone Receptor-Positive (PR+)

The presence of PR on the cell surface can be used to identify breast cancer that is PR+. Progesterone, a hormone that commonly affects breast tissue growth and development, activates these receptors. Because both subtypes frequently react to comparable hormone-targeted therapy, PR+ breast cancer is frequently considered alongside estrogen receptor-positive (ER+) breast cancer [26].

Progesterone Receptor Function

Progesterone receptors are found on cancer cells in PR+ breast cancer subtypes, rendering them susceptible to hormonal treatments. Tamoxifen and aromatase inhibitors are examples of hormone-targeted medicines that are essential to the treatment of PR+ cancerous cells and have a significant influence on patient outcomes. Progesterone receptors, which are nuclear hormone receptors, regulate cell division and proliferation in healthy breast tissue. When breast cancer cells express PRs, progesterone can impact them, causing them to respond and perhaps increase growth. To determine the PR status, which is often stated as positive (PR+) or negative (PR-), tumor tissue is immunohistochemically analyzed. ER+ and PR+ are present in 60-70% of breast cancer cases. Because hormone-targeted therapy can help reduce cancer development, these tumors often have a better prognosis than ER- and PR- cancers [27]. To make educated decisions, patients must discuss their individual diagnoses and treatment plans with their healthcare team. The cornerstone of therapy for PR+ breast cancer is hormone therapy, which covers various medicine types, as seen below in Table 1.

**Table 1:** The therapy for PR+ breast cancer is hormone therapy The authors created the table showing the treatment of PR+ breast cancer. PR: progesterone receptor; ER: estrogen receptor; SERM: selective estrogen receptor modulator.

Drugs name		PR+ treatment for breast cancer
1	Tamoxifen	SERM prevents breast cancer cells from expressing PR or other estrogen receptors.
2	Aromatase inhibitors	Inhibit the aromatase enzyme, which transforms testosterone into estradiol in subcutaneous tissue.
3	Fulvestrant	An estrogen receptor down-regulator and degrader that targets ER.

Prognosis and Therapy Response

Knowing the patient's PR status is crucial to forecasting how they will respond to hormone therapy and fare in general. On PR+ tumors, hormone-targeted treatment typically has beneficial effects, which can lead to better outcomes. ER and PR positivity is often linked with a more substantial response to hormonal therapy. The menopausal status, patient performance status, patient's comorbidities, and individual choice. Influence the selection of hormone therapy.

HER2+

The presence of HER2 receptor overexpression in breast cancer is characterized by an excess of proteins, gene amplification, or a combination of both, and it is observed in approximately 25-30% of breast cancer cases. Irrespective of the disease stage, HER2 receptor overexpression strongly correlates with poorer prognostic outcomes regarding disease-free survival and overall survival. Furthermore, it is associated with a more aggressive tumor phenotype without targeted HER2-directed therapies [28].

Specialized Treatments

One of the first targeted treatments for HER2+ was trastuzumab (Herceptin). It is a monoclonal antibody that has improved patient outcomes and targets HER2-positive cells. Trastuzumab-based combination medicines have evolved into the norm for medical treatment. Trastuzumab and chemotherapy are combined with another monoclonal antibody called pertuzumab (Perjeta) to treat HER2-positive breast cancer. It has demonstrated effectiveness in clinical studies and targets a distinct area of the HER2 receptor. Trastuzumab is combined with a chemotherapeutic medication as the antibody-drug combination ado-trastuzumab emtansine (T-DM1, Kadcyla) [29]. It has demonstrated efficiency in treating HER2+ cancerous cells [30].

More Recent Targeted Therapies

In advanced HER2+, the use of the small molecule tyrosine kinase inhibitor tucatinib (brand name Tukysa), when used together with trastuzumab and capecitabine, has received approval. Patients with brain metastases benefit most from it. Individuals diagnosed with early-stage HER2+ are given neratinib (Nerlynx), another tyrosine kinase inhibitor, in the prolonged adjuvant context after trastuzumab-based treatment. In ongoing clinical investigations, novel therapies for HER2-positive breast cancer are being investigated. Recent antibody-drug conjugates, bispecific antibodies, and HER2 immunotherapies may fall within this category [21,31].

Immunotherapy

In clinical studies, immune checkpoint inhibitors like pembrolizumab and atezolizumab are being investigated with HER2-targeted treatments. These combinations improve the ability of the immune system to target HER2-positive tumor cells [32]. Some patients may still have residual disease after receiving neoadjuvant therapy. To improve outcomes for these people, techniques including surgery, radiation treatment, and other systemic therapies are being researched.

The Function of Monoclonal Antibodies in Breast Cancer Therapy With HER2+

Pertuzumab has lately gained approval for use as an adjuvant. Pertuzumab is a monoclonal antibody that targets HER2 and functions in a way that complements the effectiveness of trastuzumab. Pertuzumab works by inhibiting heterodimerization her2-4 while trastuzumab blocks her2-2 [33,34]. An effective technique for adjuvant treatment is dual anti-HER2 blocking with trastuzumab + pertuzumab. A non-insignificant percentage of patients have a disease relapse despite the survival benefit of adding trastuzumab to early breast cancer therapy [35]. This is something that needs more research and development in the coming years.

ER+ and recent treatment

Targeting only estrogen receptors is a crucial part of treating ER+ breast cancer. With a focus on enhancing results and reducing side effects, there have been considerable advancements in treating ER+ breast cancer in recent years.

Hormone Treatment

Since hormone treatment explicitly targets the estrogen receptors on cancer cells, it is crucial in controlling cancerous cells of ER+ breast tumors. There are two primary categories of hormonal therapy.

Tamoxifen: It is a popular SERM and has consistently been the gold standard for ER+ treatment. Tamoxifen is effective by competitively interacting with the estrogen receptors on cancer cells, preventing estrogen attachment, and inhibiting cell division. Several clinical studies have supported its efficiency in lowering the chances of re-occurrence and boosting survival [36].

Aromatase inhibitors (AIs): Postmenopausal women are the main users of AIs like anastrozole, letrozole, and exemestane. They function by preventing the aromatase enzyme, which turns androgens into estrogen. By lowering estrogen levels, AIs efficiently deprive ER+ cancer cells of their growth signal. Clinical trials have seen improved outcomes and decreased recurrence rates with AI treatment [37].

Applied Therapies

CDK4/6 inhibitors: The creation and approval of CDK4/6 inhibitors, such as palbociclib, ribociclib, and abemaciclib, marks a substantial step in treating ER+ breast cancer. These drugs target CDK4 and CDK6, which are essential cell cycle regulators. CDK4 and CDK6 have shown significant progress in inhibiting metastasis in individuals with metastatic ER+ breast cancer and are now in the adjuvant setting [18].

Inhibitors of Immune Checkpoints

Immune checkpoint drugs, including pembrolizumab, have altered how some malignancies are treated, while research on how they affect ER+ breast cancer is ongoing. Early research indicates that immunotherapy and other therapies may benefit some subclass of ER+ breast cancer individuals with substantial levels of tumor mutational load. Clinical trials are still being conducted to learn more about these treatments' potential.

The Use of Genomic Profiling

Oncotype DX and MammaPrint are two genomic profile assays that have become essential resources to help understand the genomic risk of relapse and likely benefit from adjuvant chemotherapy for patients with early localized ER+ HER2-negative breast cancer. To assess the likelihood of a recurrence and the possible benefits of treatment, these tests examine the expression of several cancer genes inside tumor tissue. Oncotype DX and MammaPrint, two readily available tests, have demonstrated good metric performances in the prediction of prognosis and treatment benefits of early-stage breast cancer, and these genomic predictors are being used in various areas of normal clinical practice [38].

Neoadjuvant and Adjuvant Treatment

Adjuvant therapy is essential to treating ER+ breast cancer. The decision between hormone treatment and chemotherapy is made depending on the size of the tumor, whether lymph nodes are affected, and the patient's preferences. Adjuvant therapy seeks to lower the chance of local and distant recurrence, whereas neoadjuvant therapy decreases tumor size, increases the chance of achieving negative surgical margins, serves as biological proof of chemosensitivity, and improves surgical and medical long-term outcomes making surgery more feasible [39].

Reducing Side Effects

To reduce side effects, novel hormone treatments with better safety profiles are being developed. The treatment strategy also includes supportive care practices, including controlling menopausal symptoms, addressing psychological well-being, and managing pain. With current research and clinical trials aiming at further improving outcomes while reducing side effects and enhancing patient quality of life, these therapy methods demonstrate how ER+ breast cancer care is changing. For individuals with ER+ breast cancer, individualized treatment strategies developed in consultation with medical oncologists are still essential [40].

Inhibition of immune checkpoints in TNBC

Immune checkpoint inhibition (ICI) therapy is now the most effective immune-based treatment for producing long-lasting responses in various malignancies. Monoclonal antibodies that target PD-1/programmed death-ligand 1 (PD-L1) have become viable tools for releasing the restraints on T cell activation [41]. The US FDA has so far approved several blocking monoclonal antibodies, such as pembrolizumab, atezolizumab, and paclitaxel for PD-1 [42-44]. Only a tiny percentage of patients experience improved survival rates due to treatment reactions to immune checkpoint blockers [45]. Therefore, there is an increasing demand for biomarkers that can predict how an ICI will respond. Additionally, only a limited number of early research investigations have examined the advantages of addressing multiple immune checkpoint pathways, such as PD-1, CTLA-4, Tim3, and Lag3 [46]. Most breast cancer research projects now concentrate on blocking the PD1/PD-L1 pathway. However, no favorable outcomes were observed in patients with HR+ [47].

Immune checkpoints as vital anti-breast cancer therapy targets

Immune checkpoints are various suppressive mechanisms inscribed on the exteriors of immune and cancerous cells and are essential for controlling the intensity and persistence of immune responses against the tumor. The ligands found on the cancer cell and their matching receptors on the CD8+ T cells cell make up these checkpoints. PDL1, CD80/CD86, major histocompatibility complex class II (MHC II), CD155, and galectin-9 (GAL9) are some ligands expressed in cancer cells. Their respective receptors on the surfaces of CD8+ T cells include PD1, CTLA4, LAG3, T-cell immune receptor with immunoglobulin (Ig) and ITIM domains (TIGIT), T cell immunoglobulin and mucin-3 (TIM3), and others [48]. Additionally, there is evidence that V-set domain T-cell activation inhibitor 1 (VTCN1), although its corresponding ligand is unknown, also has a significant tumor immunosuppressive impact. Inhibitory ligand-receptor interactions are necessary for the immunological checkpoints to be activated. PD1, PDL1, and CTLA4 are the three most significant checkpoint molecules presently utilized for therapeutic development (Table 2) [49].

**Table 2:** Summaries of the articles included in the review The authors created the above table illustrating the summaries of various articles used in this review. HR: hormone receptor; PR: progesterone receptor; ER: estrogen receptor; PD-1: programmed cell death protein 1.

Sr. No.	Author’s name	Year of publication	Journal	Summary
1.	Ye F et al. [1]	2023	Molecular Cancer	Despite ongoing scientific progress, breast cancer remains a major and increasingly common health concern among women, now ranking as the second most prevalent cancer. Incidence rates have notably increased over the past four decades.
2.	Li Y et al. [2]	2022	Frontiers in Oncology	In 2020, approximately 685,000 new global breast cancer cases were reported, marked by regional variations. Higher breast cancer mortality rates were observed in economically developed countries. Projections suggest that by 2040, over 1 million will die from breast cancer, while over 3 million will be diagnosed.
3.	Baselga J et al. [12]	2012	The New England Journal of Medicine	Breast cancer's complexity, based on receptor profiles and clinical traits, poses a challenge to researchers and clinicians. Customized medicines, like Herceptin and Perjeta, have essentially promoted outcomes for HER2-positive breast cancer patients, minimizing side effects and enhancing their quality of life.
4.	Datta J et al. [17]	2022	Frontiers in Oncology	CDK4/6 inhibitors like abemaciclib, palbociclib, and ribociclib, have reduced breast cancer recurrence and improved survival, marking a notable advancement in HR+ breast cancer treatment.
5.	Hortobagyi GN et al. [18]	2016	The New England Journal of Medicine	The development and endorsement of CDK4/6 inhibitors like palbociclib, ribociclib, and abemaciclib represent a vital advancement in ER+ breast cancer treatment. These drugs specifically target CDK4 and CDK6, crucial regulators of the cell cycle. They have demonstrated substantial efficacy in impeding metastasis for individuals with metastatic ER+ breast cancer, and are now also being used in the adjuvant setting.
6.	Burguin A et al. [21]	2021	Journal of Personalized Medicine	Genomic profiling enables the detection of genetic changes driving breast cancer. This advances personalized therapy, such as using PARP inhibitors for BRCA-mutated breast cancer.
7.	Robson M et al. [22]	2017	The New England Journal of Medicine	While there have been positive advancements in targeted therapy for breast cancer, the persistent challenge lies in combating inherent and acquired therapy resistance.
8.	Purdie CA et al. [26]	2014	British Journal of Cancer (BJC)	Cell surface PR presence identifies PR+ breast cancer, influenced by progesterone, impacting breast tissue growth. PR+ is often treated like ER+ due to shared hormone-targeted therapy reactivity.
9.	Osborne CK et al. [27]	2011	Annual Review of Medicine	PR status (positive, PR+; negative, PR-) in breast cancer is determined through immunohistochemical analysis. ER+ and PR+ are found in 60-70% of cases, with better prognosis due to hormone-targeted therapy. PR status helps predict a patient's response to hormone therapy.
10.	Burstein HJ et al. [37]	2011	Journal of Clinical Oncology	Aromatase inhibitors inhibit the aromatase enzyme, which turns androgens into estrogen. This reduction in estrogen effectively hinders the growth signal for ER+ cancer cells, leading to improved clinical trial outcomes and lower recurrence rates.
11.	Moo TA et al. [39]	2018	Pet Clinics	Adjuvant therapy for ER+ breast cancer hinges on tumor size, lymph node involvement, and patient preferences, with hormone therapy or chemotherapy as options. It reduces local and distant recurrence risk.
12.	von Minckwitz G et al. [40]	2019	The New England Journal of Medicine	Current research and clinical trials aim to enhance ER+ breast cancer care by improving outcomes, reducing side effects, and improving patient quality of life. Individualized treatment strategies, in consultation with medical oncologists, remain essential for those with ER+ breast cancer.
13.	Cogdill AP et al. [46]	2017	British Journal of Cancer (BJC)	A small fraction of patients benefit from immune checkpoint blocker treatment, highlighting the need for predictive biomarkers. Limited research has explored the advantages of targeting multiple immune checkpoint pathways like PD-1, CTLA-4, Tim3, and Lag3.

## Conclusions

The landscape of breast cancer targeted therapy has changed substantially over time, giving patients new chances and optimism. Breast cancer's numerous subtypes need specialized treatment strategies, and it continues to be a significant worldwide health concern. A new age of precision medicine has begun due to targeted therapeutics, enabling more efficient and less toxic treatments. Targeted medications have entirely changed how breast cancer is treated due to the discovery of particular molecular markers. Immunotherapies, monoclonal antibodies, and small molecule inhibitors have significantly enhanced patient outcomes. These medicines more precisely target cancer cells and offer less toxic alternatives than regular chemotherapies. Genomic profiling allows for more individualized treatment plans, which aids clinicians in making informed decisions and prevents patients from obtaining unnecessary treatments. Nevertheless, difficulties persist, notably in comprehending and removing resistance mechanisms and guaranteeing equal access to these treatments. This thorough analysis of ongoing studies and clinical trials shows hope for future improvements in breast cancer treatment. New medications and cutting-edge methods, such as immune checkpoint inhibitors and combination therapy, show promise for enhancing outcomes, particularly in complex subtypes like TNBC. We may work toward more individualized and successful therapies by remaining on top of the most recent advancements in targeted therapy, which will enable us to significantly advance our fight against this challenging condition.
